# Oncogenic Akt-FOXO3 loop favors tumor-promoting modes and enhances oxidative damage-associated hepatocellular carcinogenesis

**DOI:** 10.1186/s12885-019-6110-6

**Published:** 2019-09-05

**Authors:** Miao Lu, Daniel Hartmann, Rickmer Braren, Aayush Gupta, Baocai Wang, Yang Wang, Carolin Mogler, Zhangjun Cheng, Thomas Wirth, Helmut Friess, Jörg Kleeff, Norbert Hüser, Yoshiaki Sunami

**Affiliations:** 1 School of Medicine, Klinikum rechts der Isar, Department of Surgery, Technical University of Munich, Munich, Germany; 2 Department of General Surgery, Zhongda Hospital, Southeast University, Nanjing, China; 3 School of Medicine, Klinikum rechts der Isar, Institute for diagnostic and interventional Radiology, Technical University of Munich, Munich, Germany; 4 Institute of Pathology, Technical University of Munich, Munich, Germany; 5 Institute of Physiological Chemistry, University of Ulm, Ulm, Germany; 6 Department of Visceral, Vascular and Endocrine Surgery, University Medical Center Halle, Martin-Luther-University Halle-Wittenberg, Halle, Germany

**Keywords:** HCC, FOXO, Akt, ROS, DNA damage, NADPH

## Abstract

**Background:**

Hepatocellular carcinoma (HCC) is the most prevalent primary liver cancer, accounting for 80–90% of cases. Mutations are commonly found in the signaling regulating the PI3K/Akt pathway, leading to oncogenic cell proliferation and survival. Key transcription factors that are negatively regulated downstream of PI3K/Akt are members of the forkhead box O family (FOXO). FOXOs were initially considered as tumor suppressors by inducing cell cycle arrest and apoptosis. However, there is increasing evidence showing that FOXOs, especially FOXO3, can support tumorigenesis.

**Methods:**

To understand the roles of FOXO3 in liver tumorigenesis and hepatocarcinogenesis, we analyzed HCC patient specimens and also established a doxycycline-regulated transgenic mouse model with hepatocyte-specific FOXO3 expression in a constitutively active form.

**Results:**

We found that FOXO3 protein is significantly overexpressed and activated in livers of HCC patients. Hepatic activation of FOXO3 induced extensive hepatic damage and elevated gene expression of several HCC-associated factors. Furthermore, FOXO3 expression enhanced hepatotoxicin-induced tumorigenesis. Mechanistically, FOXO3 activation caused oxidative stress and DNA damage and triggered positive feedback-loop for Akt activation as well as mTORC2 activation. Interestingly, FOXO3 activated not only reactive oxygen species (ROS)-promoting pathways, but also ROS-eliminating systems, which can be associated with the activation of the pentose phosphate pathway.

**Conclusions:**

FOXO3 is a master regulator of ROS in a ‘carrot and stick’ manner; on one side avoiding cellular crisis while also supporting hepatocellular carcinogenesis. Clinically, we suggest analyzing FOXO3 activation status in patients with liver diseases, in addition to PI3K/Akt signaling. Personalized therapy of FOXO3 inhibition may be a reasonable, depending on the activation status of FOXO3.

## Background

Primary liver cancer is the fifth most common cancer worldwide and the second leading cause of cancer-related death worldwide [1]. Hepatocellular carcinoma (HCC) is the most prevalent primary liver cancer and accounts for 80–90% of cases. Chronic infection with hepatitis B virus (HBV) remains the leading cause of HCC, but other etiologies, including chronic infection with hepatitis C virus (HCV), chronic alcohol abuse, α-1 antitrypsin deficiency, as well as hemochromatosis, are known causes of HCC. Sustained cell death and regeneration, cellular stress and mitochondrial alteration can promote tumorigenesis, where survival and proliferation signals are dysregulated [2]. Mutations are commonly found in signaling regulating the PI3K/Akt pathway, leading to oncogenic cell proliferation and survival [3–5]. Also in HCC, the PI3K/Akt cascade plays a central position in the signaling pathway network [4].

Key transcription factors that are negatively regulated downstream of PI3K/Akt are members of the forkhead box O family (FOXO). The FOXO group contains four members: FOXO1 (FKHR), FOXO3 (FKHRL1), FOXO4 (AFX), and FOXO6 [6]. Akt-mediated phosphorylation of FOXO transcription factors inhibits their function by enhancing interaction with chaperones and exporting them from the nucleus to the cytoplasm (except for FOXO6) [6, 7]. Active FOXO transcription factors regulate the expression of a diverse array of cellular genes involved in redox homeostasis, cell proliferation, differentiation, metabolism and apoptosis [8, 9]. As FOXOs are effectors that are negatively regulated downstream of Akt signaling, they were initially considered as tumor suppressors because of their influence on cell cycle arrest and apoptosis [5]. However, there is increasing evidence showing that FOXOs, especially FOXO3, can support tumorigenesis in several cancer types: High expression of FOXO3 is associated with glioblastoma progression and prediction of poor survival of patients [10]. Pancreatic cancer patients with high FOXO3 activation signatures show a shorter overall survival rate than patients with low FOXO3 activation [11]. Recent publication suggests that FOXO3 can both suppress and support breast cancer progression [12].

Whether FOXO3 suppresses or supports HCC has not been investigated. Hence, in the current study we first analyzed FOXO3 expression and activation status in HCC patients. Histologically, we observed that FOXO3 protein is overexpressed and activated in livers of HCC patients. To see whether hepatic activation of FOXO3 can sufficiently support hepatocellular carcinogenesis, or whether FOXO3 activation is just a secondary-effect during tumor development, we further generated a mouse model with hepatocyte-specific expression of a constitutively active form of FOXO3. Constitutive activation of FOXO3 led to extensive hepatic damage and enhanced hepatotoxicity-induced tumorigenesis. On the one hand, extensive liver damage is presumably associated with FOXO3-mediated ROS pathway activation. On the other hand, constitutively active FOXO3 expression can also induce counteraction against ROS and activation of Akt.

## Methods

### Human subjects

Human liver samples from patients with HCC or an area of non-HCC were obtained from the Department of Surgery, Klinikum rechts der Isar, Technical University Munich, Germany. The study on human material was approved by the institutional review board of the Medical Faculty of the Technical University Munich and designed in accordance with the Declaration of Helsinki (Approval number: 5846/13). Written informed consent was obtained from patients.

### Animal experimentation

We crossed mice expressing the tetracycline-responsive transactivator (tTA) under the control of the rat liver-enriched transcriptional activator protein (LAP) promotor [13, 14] with mice bearing a constitutively active human *FOXO3* allele [15, 16] under the control of a tTA-regulated promotor. FOXO3 transgenic mice have been generated in house, and tTA animals were originally obtained from TET Systems GmbH (Heidelberg, Germany) with the material transfer agreement. All mice were on a C57BL/6 and NMRI-mixed background, resulting in heterozygous FOXO3CA^Hep^ mice in mixed gender. Control animals were sibling littermates. Mice were fed with standard diet from Altromin (1324, maintenance diet for rats and mice), bedded with select fine (Rettenmaier & Söhne), enriched with reusable red polycarbonate houses, disposable cellulose houses, wood bricks, and cotton fiber nestlets in individually ventilated cages. Diethylnitrosamine (DEN)-injection was intraperitoneally performed during the light cycle only with male mice at the age of 15 days (10 mg / kg body weight, DEN was dissolved in 0.9% NaCl). Transgenic, constitutively active FOXO3 expression was repressed by doxycycline administration in the drinking water (0.1 g / l) until the age of 5 weeks. For mechanistic characterization, 9-week-old FOXO3CA^Hep^ transgenic mice (transgene expression for 4 weeks) as well as sibling littermates were analyzed. For analyzing the effect of FOXO3 for tumorigenesis (e.g. tumor incidence, tumor numbers), we analyzed 40-week-old male mice in 4 groups: control, FOXO3CA^Hep^, DEN-injected control and DEN-injected FOXO3CA^Hep^ mice. For each group, we used maximum 6 animals. All mice were genotyped and the experiments were not randomized. Anaesthesia (for Magnetic Resonance Imaging (MRI) and organ harvesting / blood withdrawal) was maintained by mask inhalation of isoflurane vaporized at concentrations of up to 4% in the initial phase and at 2% during imaging or surgical procedure. It was saturated with 36% F(i)O2. During isoflurane anaesthesia, no breathing complications were observed. As a buprenorphine based regimen for the treatment of surgical pain, Temgesic (0.05 mg/kg body weight) was given subcutaneously and mice were administered Metamizole (200 mg/kg body weight) orally before skin incision. At the end of the particular experiment, mice were anaesthetized under isoflurane inhalation and exsanguination was induced by a puncture of the abdominal aorta with needle and syringe.

Animal experiments and the proposal were designed according to the European and German laws (Tierschutzgesetz) and institutionally approved by the District Government of Upper Bavaria in Germany (ref: 55.2–1-54-2532-164-2014).

### Immunohistochemistry

**Fig. 1:**
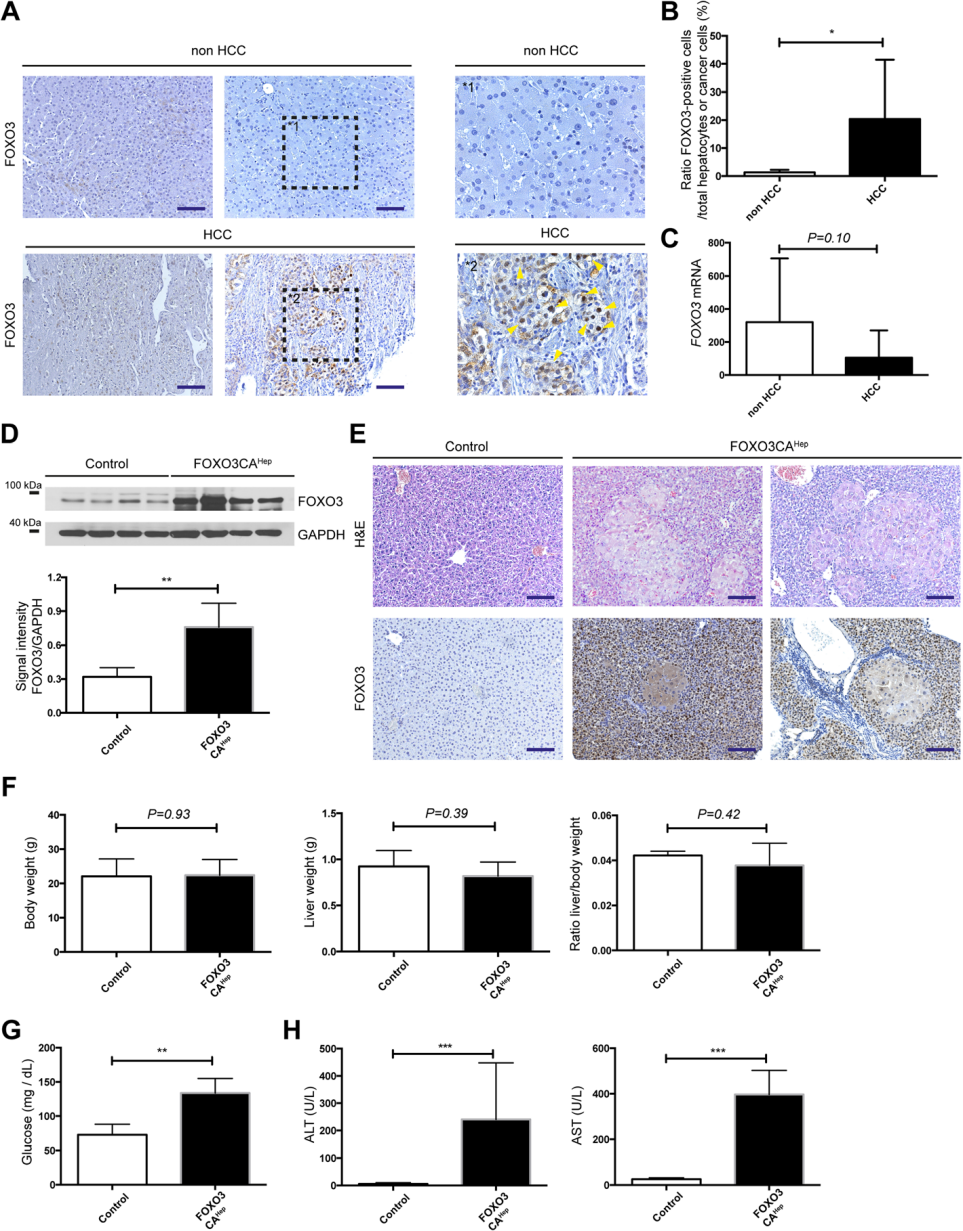
FOXO3 signaling is activated in hepatocellular carcinoma patients, and hepatocyte-specific activation of FOXO3 causes extensive hepatocyte damage. **a** Detection of activated FOXO3 in livers of patients in HCC or non-HCC groups (left and middle panels) and magnification (right panel) as determined by FOXO3 immunohistochemistry (bar = 100 μm). **b** Quantification of FOXO3-positive cells as a percentage of total hepatocytes/cancer cells in livers of patients in HCC (*n* = 27) or non-HCC groups (*n* = 6). **c** Expression of FOXO3 by qPCR in livers of patients in HCC or in non-HCC groups (*n* = 11). **d** Western blot analysis in livers from 9-week-old control and FOXO3CA^Hep^ mice with FOXO3 and GAPDH as loading control (upper panel), as well as densitometric quantification (lower panel). **e** Representative hematoxylin and eosin and IHC staining for FOXO3 (bar = 100 μm). **f** Body weight and liver weight of 9-week-old control and FOXO3CA^Hep^ transgenic mice (*n* = 4). **g** Blood glucose levels were measured from control and FOXO3CA^Hep^ transgenic mice. **h** Alanine amino transferase and aspartate amino transferase levels were elevated in 9-week-old FOXO3CA^Hep^ versus control mice

Liver specimens were fixed in 4% PFA and embedded in paraffin. These paraffin blocks were cut into 2.5 μm sections for immunohistochemistry staining. Briefly, the prepared sections were deparaffinized using Roticlear and rehydrated with a descending alcohol row. Then these slides were microwaved in citrate buffer (pH 6.0; 10 mM Citric Acid) for 15 min for antigen retrieval. Afterwards, these sections were incubated in 3% hydrogen peroxide to quench the endogenous peroxidase, and in 3% bovine serum albumin (Carl Roth) (for phosphor-Akt (Ser 473), 10% goat serum (for Collagen IV, E-Cadherin, N-Cadherin, FOXO3, Ki67, p53, Vimentin), or 10% donkey serum (for Alpha-Fetoprotein (AFP)) for antigen blocking. An addition of 0.3% Triton-X 100 (Carl Roth) was used to break the cell membrane before blocking for p-Akt and p53 staining. The sections were subsequently incubated with primary antibodies at 4 °C overnight with appropriate secondary antibodies. Finally, signals were detected using DAB+ or AEC+ (DAKO). The antibodies used for immunohistochemical experiments in this study are as follows:

AFP (R&D, AF5369, Goat, dilution 1:200), phospho-Akt (Ser 473) (Cell Signaling, 4060, Rabbit, dilution 1:50), Collagen IV (Abcam, ab6586, Rabbit, dilution 1:200), E-Cadherin (Cell Signaling, 3195, Rabbit, dilution 1:400), N-Cadherin (Cell Signaling, 13,116, Rabbit, dilution 1:125), FOXO3 (Santa Cruz, H144, sc-11,351, Rabbit, dilution 1:200 for Fig. 1, Cell Signaling, 12,829, Rabbit, 1:3000 for Fig. 3), phospho-γH2AX (Novus Biologicals, NB100–2280, Rabbit, dilution 1:200), 8-Hydroxyguanosine (Abcam, ab48508, Mouse, dilution 1:3000), Ki67 (Abcam, 16,667, Rabbit, dilution 1:200), p53 (Leica, NCL-L-p53-CM5p, Rabbit, dilution 1:200), Vimentin (Abcam, ab92547, Rabbit, dilution 1:200).

For quantification of stainings of phospho-γH2AX, 8-Hydroxyguanosine and Ki67, staining-positive cells as well as total hepatocytes/cells were counted in 4 random high-power fields of liver tissue for each animal and obtained values were merged for further statistical analysis.

### Western blotting and qPCR experiments

Western blotting and qPCR experiments were performed as previously described [13, 14]. The antibodies and primers used in this study are as follows:

Antibodies: AFP (R&D, AF5369, Goat, dilution 1:200), Akt (Cell Signaling, 9272, Rabbit, dilution 1:1000), phospho-Akt (Ser 473) (Cell Signaling, 4060, Rabbit, dilution 1:1000), E-Cadherin (Cell Signaling, 3195, Rabbit, dilution 1:1000), N-Cadherin (Cell Signaling, 13,116, Rabbit, dilution 1:1000), FOXO3 (Cell Signaling, 12,829, Rabbit, dilution 1:1000), GAPDH (Cell Signaling, 2118, Rabbit, dilution 1:5000), MSH2 (Cell Signaling, 2017, Rabbit, dilution 1:1000), c-Myc (Abcam, ab32072, Rabbit, dilution 1:1000), p53 (Cell Signaling, 2524, Mouse, dilution 1:1000), Rictor (Cell Signaling, 2114, Rabbit, dilution 1:1000), Vimentin (Cell Signaling, 5741, Rabbit, dilution 1:1000).

Primers: *FOXO3* (human) Forward: CTTCAAGGATAAGGGCGACA Reverse: CGACTATGCAGTGACAGGTTG, *GAPDH* (human) Forward: GGAGCGAGATCCCTCCAAAAT Reverse: GGCTGTTGTCATACTTCTCATGG, *Gapdh* (mouse) Forward: AGGTCGGTGTGAACGGATTTG Reverse: TGTAGACCATGTAGTTGAGGTCA, *Myc* (mouse) Forward: ATGCCCCTCAACGTGAACTTC Reverse: CGCAACATAGGATGGAGAGCA, *Trp53* (mouse) Forward: CTCTCCCCCGCAAAAGAAAAA Reverse: CGGAACATCTCGAAGCGTTTA, *Kras* (mouse) Forward: CAAGAGCGCCTTGACGATACA Reverse: CCAAGAGACAGGTTTCTCCATC, *Afp* (mouse) Forward: CTTCCCTCATCCTCCTGCTAC Reverse: ACAAACTGGGTAAAGGTGATGG, Msh2 (mouse) Forward: GTGCAGCCTAAGGAGACGC Reverse: CTGGGTCTTGAACACCTCGC, *Bcl2l11* (Bim, mouse) Forward: GACAGAACCGCAAGGTAATCC Reverse: ACTTGTCACAACTCATGGGTG, *Gpx1* (mouse) Forward: AGTCCACCGTGTATGCCTTCT Reverse: GAGACGCGACATTCTCAATGA, *Gpx2* (mouse) Forward: GCCTCAAGTATGTCCGACCTG Reverse: GGAGAACGGGTCATCATAAGGG, *Gpx3* (mouse) Forward: CCTTTTAAGCAGTATGCAGGC Reverse: CAAGCCAAATGGCCCAAGTT, *Sesn2* (mouse) Forward: TCCGAGTGCCATTCCGAGAT Reverse: TCCGGGTGTAGACCCATCAC, *Sesn3* (mouse) Forward: CGGAAGGACAAAAGAATCCGA Reverse: GTTCATCCGCCGTATTTGCT, *G6pdx* (mouse) Forward: CACAGTGGACGACATCCGAAA Reverse: AGCTACATAGGAATTACGGGCA.

### MRI imaging

Tumor growth kinetics in mice were followed by T2-weighted (T2w) MRI using an 8 channel wrist coil and a 3.0 Tesla clinical scanner (Philips), as previously described [17]. In brief, an axial T2w TSE sequence (resolution 0.3 × 0.3 × 0.7 mm^3^, minimum 30 slices, TE = 90 ms, TR > 3 s) was applied for tumor detection. Tumor volumes were calculated using Osirix. MRI imaging was initiated at an age of 40 weeks. Before imaging, mice were anaesthetized by continuous gaseous infusion of 2% isoflurane (Abbott) using a veterinary anaesthesia system (Vetland Medical). During imaging, the dose was kept at a constant 2% isoflurane, the animals’ temperature was maintained and eyes were protected with a lubricating eye ointment.

### Enzyme-linked immunosorbent assay (ELISA)

The serum level of AFP was quantified with the mouse AFP Quantikine ELISA kit (R&D, MAFP00). Experiments were performed as described by the manufacturer.

### Blood glucose and transaminase measurements

Blood glucose levels were measured with Free Style Precision Blood Glucose test strips (Abbott) directly after recovering blood. Levels of transaminase were measured with blood serum by Cobas 8000 modular analyzer (Roche). Serum was recovered from blood with Serum-Gel Z (Sarstedt) by centrifuging at 4487 g for 6 min at 4 °C.

### NADPH assay

NADPH/NADP+ ratio was measured using a NADP/NADPH assay kit (Abcam, ab65349). Briefly, approximately 50 mg of frozen liver tissue samples were homogenized in the NADP/NADPH extraction buffer supplied in the kit. Then samples were centrifuged at full speed for 5 min and the supernatants were collected in new tubes. Enzymes, which can consume NAPDH, were removed by using a 10 kD Spin Column (Abcam, ab93349). Afterwards, all samples were separated into two parts (one part for the measurement of total NADP and NADPH, and the other part for the measurement of only NAPDH). To measure NADPH, samples were incubated at 60 °C for 30 min. The heated and unheated samples were used to detect NADPH, and total NADP and NADPH, by setting up a reaction cycle according to the manufacturer’s instructions. The values were read by testing the absorbance at 450 nm and the NADPH/NADP+ ratio was calculated.

### Statistical analysis

Statistical analysis was performed using GraphPad Prism software, version 6.0 (GraphPad Software). Data are shown as means ± standard error of the mean, if not indicated differently. Statistical significance was determined using a two-tailed Student’s *t* test. *P* < 0.05 was considered significant (*P < 0.05, **P < 0.01, ***P < 0.001).

## Results

### FOXO3 signaling is activated in liver parenchymal cells of hepatocellular carcinoma patients

First of all, we addressed the question of whether FOXO3 is highly expressed and/or activated in human HCC specimens. We therefore performed RT-qPCR analyses as well as histological experiments for FOXO3. Although we did not observe any significant difference of FOXO3 mRNA expression between livers from HCC patients and non-HCC patients (*n* = 11, *P* = 0.10) (Fig. 1c), we observed FOXO3-positivity in HCC patients mainly in cancer cells but not in stroma, where FOXO3 was localized in the nucleus (Fig. 1a, b). These data suggested that FOXO3 protein is significantly overexpressed (*P* < 0.05) and activated in livers of 27 HCC-patients compared to livers from 6 non-HCC patients (Fig. 1a-c).

### Hepatocyte-specific activation of FOXO3 causes extensive hepatocyte damage and overexpression of cancer-associated genes

**Fig. 2:**
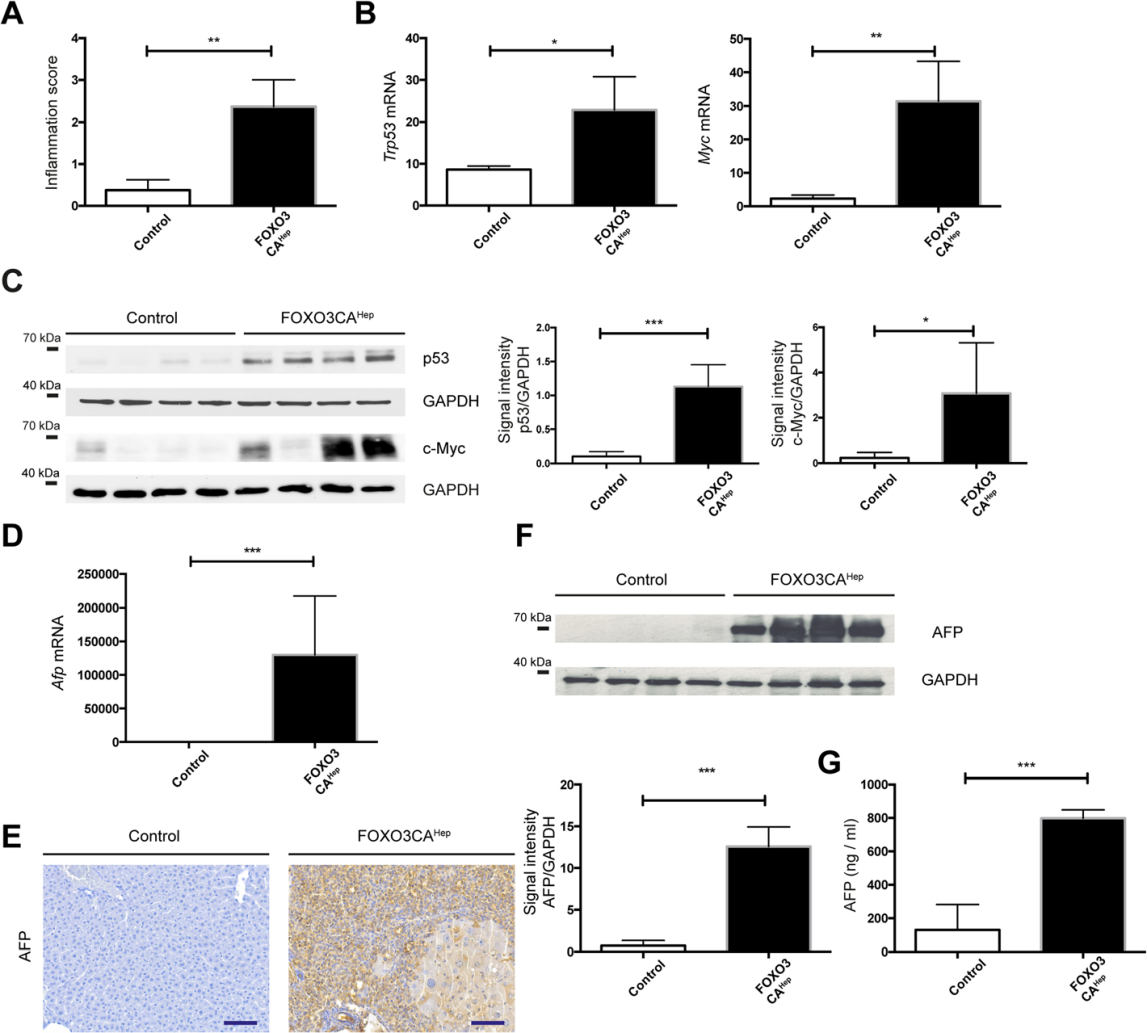
Extensive hepatocyte damage by hepatocyte-specific activation of FOXO3 associated with upregulation of HCC-concomitant factors. **a** More inflammation can be observed in livers from 9-week-old FOXO3CA^Hep^ transgenic mice than in control animals. **b** Expression of *Trp53* and *Myc* by qPCR in livers from 9-week-old control and FOXO3CA^Hep^ transgenic mice. **c** Western blot analysis in livers from 9-week-old control and FOXO3CA^Hep^ mice with p53 and c-Myc and GAPDH as loading control (left panel), as well as densitometric quantification (middle and right panels). **d** Expression of *Afp* by qPCR in livers from 9-week-old control and FOXO3CA^Hep^ transgenic mice (*n* = 4). **e** Representative IHC staining for AFP (bar = 100 μm).**f** Western blot analysis in livers from 9-week-old control and FOXO3CA^Hep^ mice with AFP and GAPDH as loading control (upper panel), as well as densitometric quantification (lower panel). **g** Serum AFP levels in 9-week-old control and FOXO3CA^Hep^ mice

On the one side, it is possible that hepatic activation of FOXO3 in patients sufficiently supports hepatocellular carcinogenesis, but on the other side it is also possible that FOXO3 activation is just a consequence and secondary-effect during tumor development. Therefore, we generated a mouse model expressing a constitutively active form of FOXO3 in hepatocytes (FOXO3CA^Hep^ mice) in order to evaluate the role of FOXO3 in hepatocellular carcinogenesis. Transgenic, constitutively active FOXO3 expression was repressed by doxycycline administration in the drinking water until the age of 5 weeks to rule out effects in liver organogenesis. Protein expression of FOXO3 in whole-liver lysates of 9-week-old FOXO3CA^Hep^ transgenic mice (transgene expression for 4 weeks) was evaluated by western blot (Fig. 1d). Interestingly, constitutive activation of FOXO3 in hepatocytes led to extensive histological and morphological alterations, exhibiting atrophic FOXO3-positive hepatocytes in combination with locally regenerated enlarged hepatocytes (Fig. 1e). We did not observe any significant change in body weight (*P* = 0.93) and liver weight (*P* = 0.39), nor in liver-to body weight ratio (*P* = 0.42) (*n* = 4), (Fig. 1f). A significant higher blood glucose level in FOXO3CA^Hep^ mice than in control animals was observed (*P* < 0.01) (Fig. 1g). We further observed an increase in liver transaminase levels (alanine aminotransferase and aspartate transaminase, *P* < 0.001) suggesting that constitutive activation of FOXO3 in hepatocytes drives extensive hepatic damage and regeneration (Fig. 1h). We further observed that hepatic activation of FOXO3 enhanced liver inflammation (Fig. 2a) and led to an elevation of cancer-associated genes, such as *Trp53* (*P* < 0.05) and *Myc* (*P* < 0.01) (Fig. 2b). We further observed elevated protein expression levels of p53 (*P* < 0.001) and c-Myc (*P* < 0.05) (Fig. 2c). FOXO3CA^Hep^ mice also showed an elevated protein and RNA expression of alpha-fetoprotein (AFP) (*P* < 0.001) (Fig. 2d-f). Further elevated AFP levels in blood serum was observed in FOXO3CA^Hep^ mice than in control animals (*P* < 0.001) (Fig. 2g).

### Hepatic activation of FOXO3 significantly promotes hepatocellular tumorigenesis

**Fig. 3:**
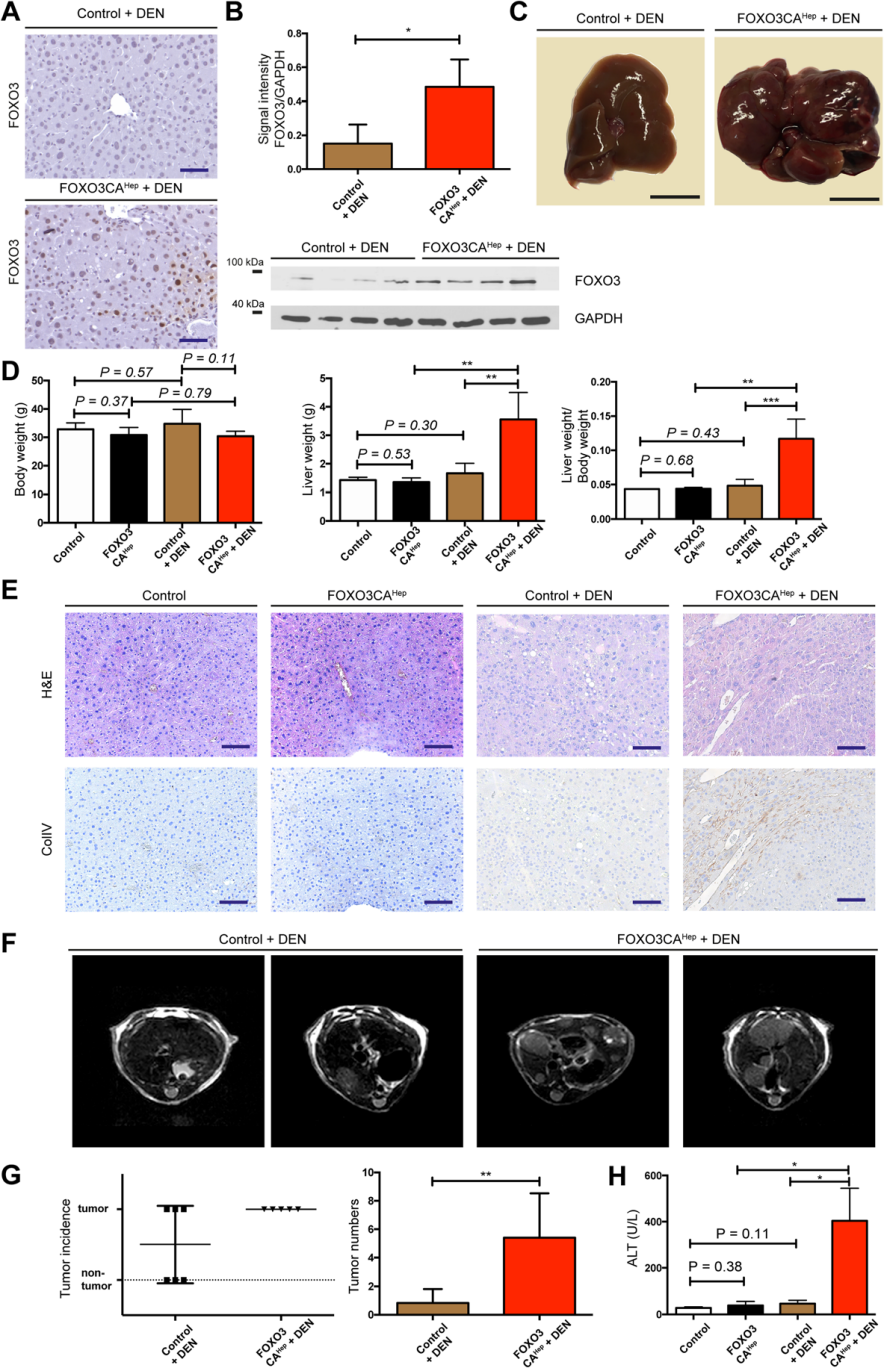
Hepatic activation of FOXO3 supports hepatotoxicity-mediated tumorigenesis. **a** Representative IHC staining for FOXO3 in 40-week-old DEN-injected control and DEN-injected FOXO3CA^Hep^ transgenic mice (bar = 100 μm). **b** Western blot analysis in livers from 40-week-old DEN-injected control and DEN-injected FOXO3CA^Hep^ transgenic mice with FOXO3 and GAPDH as loading control (lower panel), as well as densitometric quantification (upper panel). **c** Macroscopic appearance of livers in DEN-injected control and FOXO3CA^Hep^ transgenic 40-week-old mice (bar = 1 cm). **d** Body weight and liver weight of 40-week-old control, FOXO3CA^Hep^, DEN-injected control and DEN-injected FOXO3CA^Hep^ transgenic mice (control and FOXO3CA^Hep^ *n* = 3, DEN-injected control and DEN-injected FOXO3CA^Hep^, *n* = 5). **e** Representative hematoxylin and eosin and IHC staining for Collagen IV (bar = 100 μm). **f** Representative MRI imaging of livers from DEN-injected control and DEN-injected FOXO3CA^Hep^ transgenic mice. **g** Tumor numbers, incidence and volume in livers from DEN-injected control (*n* = 6) and DEN-injected FOXO3CA^Hep^ transgenic mice (*n* = 5). **h** Alanine aminotransferase levels in 40-week-old FOXO3CA^Hep^ control, FOXO3CA^Hep^, DEN-injected control and DEN-injected FOXO3CA^Hep^ transgenic mice (*n* = 3)

**Fig. 4:**
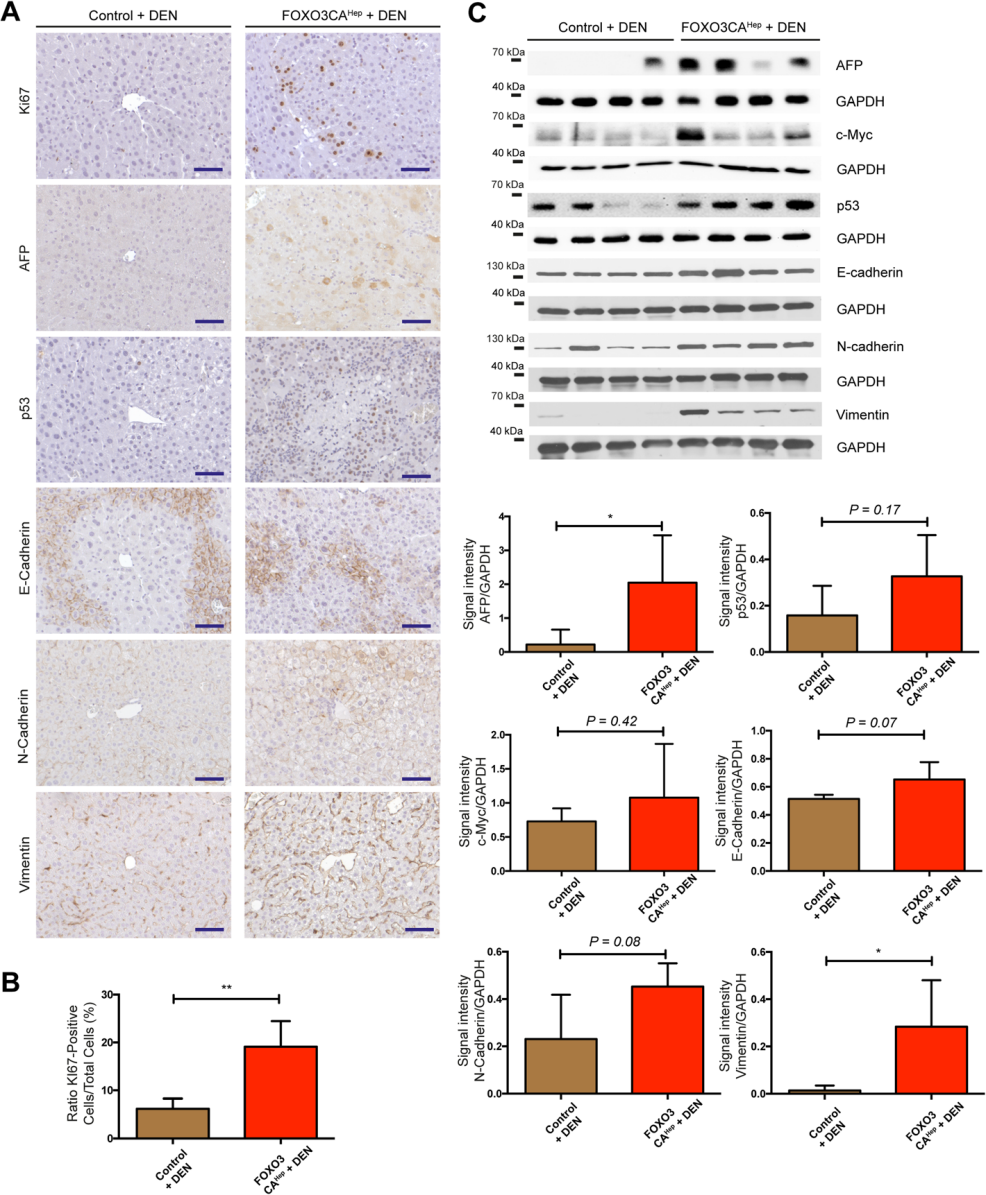
Enhanced proliferation and elevated expression of AFP and Vimentin in livers of DEN-injected 40-week-old FOXO3CA^Hep^ transgenic mice. **a** Representative IHC staining for Ki67, AFP, p53, E-Cadherin, N-Cadherin, and Vimentin in 40-week-old DEN-injected control and DEN-injected FOXO3CA^Hep^ transgenic mice (bar = 100 μm). **b** Scoring of Ki67-positivity (*n* = 4). **c** Western blot analysis in livers from 40-week-old DEN-injected control and DEN-injected FOXO3CA^Hep^ transgenic mice with AFP, c-Myc, p53, E-Cadherin, N-Cadherin, Vimentin and GAPDH as loading control (upper part), as well as densitometric quantification (lower part)

Since constitutive activation of FOXO3 in hepatocytes caused extensive tissue damage, we hypothesized that FOXO3 can support hepatotoxicity-mediated hepatocellular carcinogenesis. Therefore, to see whether hepatic activation of FOXO3 can enhance DEN-mediated hepatocellular carcinogenesis, we injected hepatotoxin diethylnitrosamine (DEN) into both control and FOXO3CA^Hep^ mice at the age of 15 days. Health status of the animals was checked daily, and we did not observe any adverse event. At the age of 40 weeks, we analyzed mice in 4 groups: control, FOXO3CA^Hep^ (*n* = 3), DEN-injected control and DEN-injected FOXO3CA^Hep^ mice (*n* = 5). Livers from DEN-injected FOXO3CA^Hep^ mice were significantly larger than livers from FOXO3CA^Hep^ mice (*P* < 0.01) or from DEN-injected control animals (*P* < 0.01) (Fig. 3a-d). Livers from DEN-injected FOXO3CA^Hep^ mice exhibited an altered collagen IV staining pattern (Fig. 3e). An increased tumor number (*P* < 0.01) with a 100% tumor incidence in 5 DEN-injected FOXO3CA^Hep^ mice compared to 6 DEN-injected control animals was observed by MRT (Fig. 3f, g). In addition, hepatocyte damage was also significantly higher in DEN-injected FOXO3CA^Hep^ mice compared to the other groups as determined by the ALT levels (*n* = 3, *P* < 0.05) (Fig. 3h). Furthermore, we observed higher expression of AFP (*P* < 0.05) as well as Vimentin (*P* < 0.05) and higher rate of proliferation rate monitored by Ki67 staining (*P* < 0.01) in DEN-injected FOXO3CA^Hep^ mice compared to DEN-injected control animals (Fig. 4). These data support our idea that hepatic activation of FOXO3 promotes DEN-induced, hepatotoxicity-mediated tumorigenesis.

### Hepatic activation of FOXO3 induces oxidative damage and Akt activation

**Fig. 5:**
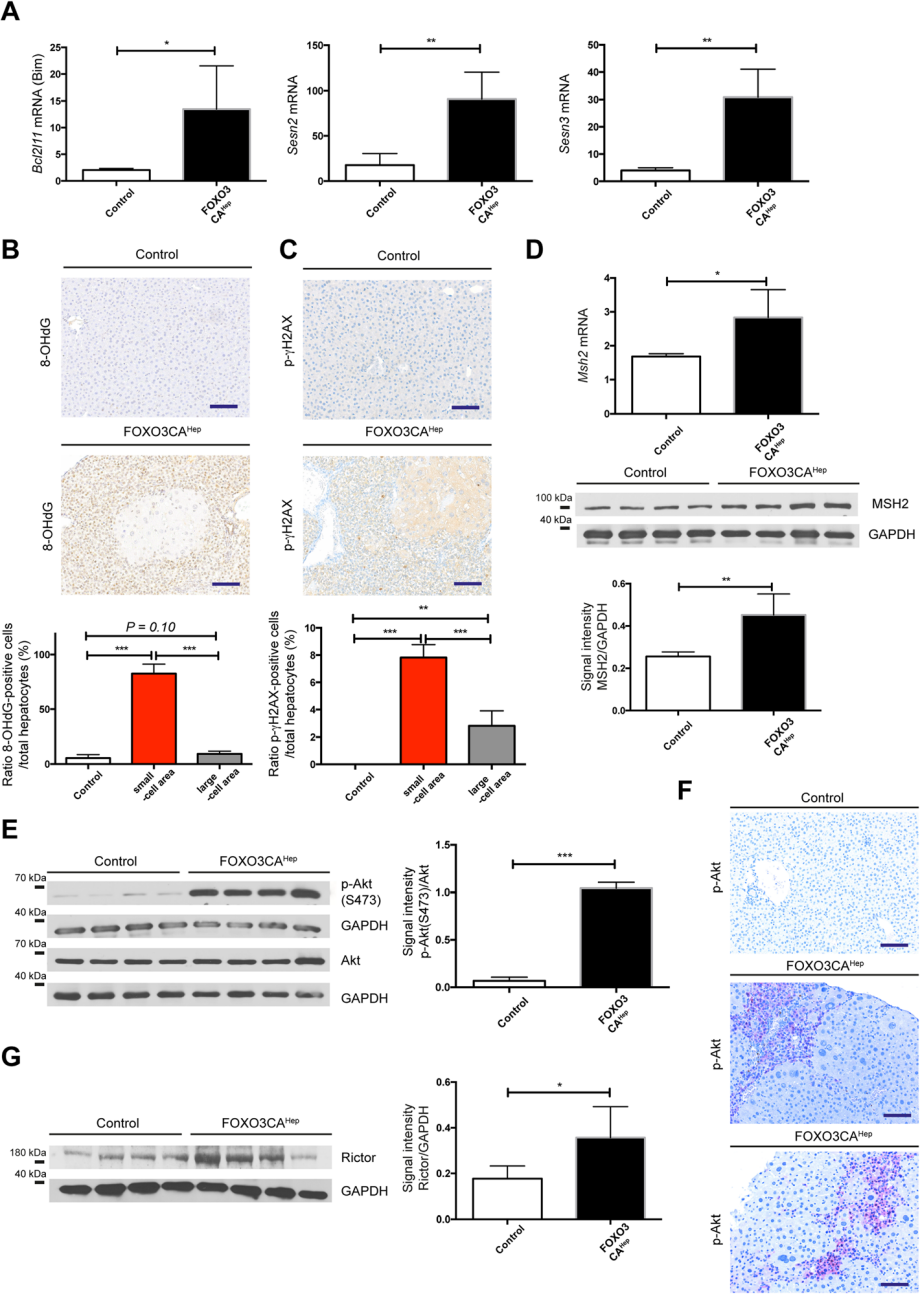
Hepatic activation of FOXO3 induces oxidative damage and Akt activation**. a** Expression of *Bcl2l11* (Bim), *Sesn2* and *Sesn3* by qPCR in livers from 9-week-old control and FOXO3CA^Hep^ transgenic mice (*n* = 4). **b** Representative IHC staining for 8-OHdG (bar = 100 μm) and quantification of 8-OHdG-positive cells as a percentage of total hepatocyte cells in livers of patients in area of small cells (SC) and large cells (LC). **c** Representative IHC staining for phospho-γH2AX (bar = 100 μm) and quantification of phospho-γH2AX-positive cells as a percentage of total hepatocyte cells in livers of patients in area of small cells and large cells. **d** Expression of *Msh2* by qPCR (upper panel), Western blot analysis for MSH2 (middle panel) and densitometric quantification (lower panel) in livers from 9-week-old control and FOXO3CA^Hep^ transgenic mice (*n* = 4). **e** Western blot analysis in livers from 9-week-old control and FOXO3CA^Hep^ mice for serine-473 phospho-Akt, with Akt and GAPDH as loading control (left panel), as well as densitometric quantification (right panel). **f** Representative IHC staining for phospho-Akt in livers from 9-week-old control and FOXO3CA^Hep^ mice (bar = 100 μm). **g** Western blot analysis in livers from 9-week-old control and FOXO3CA^Hep^ mice for Rictor and GAPDH as loading control (left panel), as well as densitometric quantification (right panel)

We next tried to identify the major causes of tissue damage due to FOXO3 overexpression. It has been shown that FOXO3 regulates the reactive oxygen species (ROS) BCL2L11 (Bim) and peroxiredoxicin SESN3 in neuronal cells [18]. We hypothesized that liver damage in FOXO3CA^Hep^ mice can be also caused by ROS and mediated by Bim. We then performed RT-qPCR experiments for *Bcl2l11* (Bim), *Sesn3*, as well as *Sesn2,* and observed a significantly higher gene expression in livers from FOXO3CA^Hep^ mice compared to control animals (*n* = 4, *P* < 0.05, *P* < 0.01, *P* < 0.01, respectively) (Fig. 5a). Markers of oxidative stress and DNA damage, 8-OHdG and serine-139 phospho-γH2AX, were significantly increased in livers of FOXO3CA^Hep^ mice, especially in atrophic cells (*P* < 0.001) (Fig. 5b, c). Expression of MSH2, an enzyme involved in DNA mismatch repair, was elevated in livers from FOXO3CA^Hep^ mice compared to control animals (*n* = 4, RNA level *P* < 0.05, protein level *P* < 0.01) (Fig. 5d). It has been shown that FOXOs activate Akt by inducing the expression of SESN3 and increased mTORC2 activity [19]. Since we observed a significantly increased expression of *Sesn3* in FOXO3CA^Hep^ mice, we speculated that hepatic activation of FOXO3 also regulates Akt in a positive-feedback manner. We performed western blotting and found that Akt was activated in livers of FOXO3CA^Hep^ mice: Ratio of serine-473 phospho-Akt/Akt signal intensity was significantly higher in FOXO3CA^Hep^ mice compared to control animals (*P* < 0.001) (Fig. 5e). Immunohistochemical analyses further showed that activation of Akt can be detectable in atrophic hepatocytes (Fig. 5f). Phosphorylation of serine-473 residue is a marker of activation of mTORC2 [20], and we observed further elevated expression of Rictor, a mTORC2-specific protein, in livers from FOXO3CA^Hep^ mice compared to control animals (*n* = 4, *P* < 0.05) (Fig. 5g). Taken together, hepatic activation of FOXO3 induces ROS and Akt activation via mTORC2 activation presumably in a cell-autonomous manner.

### Hepatic activation of FOXO3 induces an oxidative damage protection system, as well

**Fig. 6:**
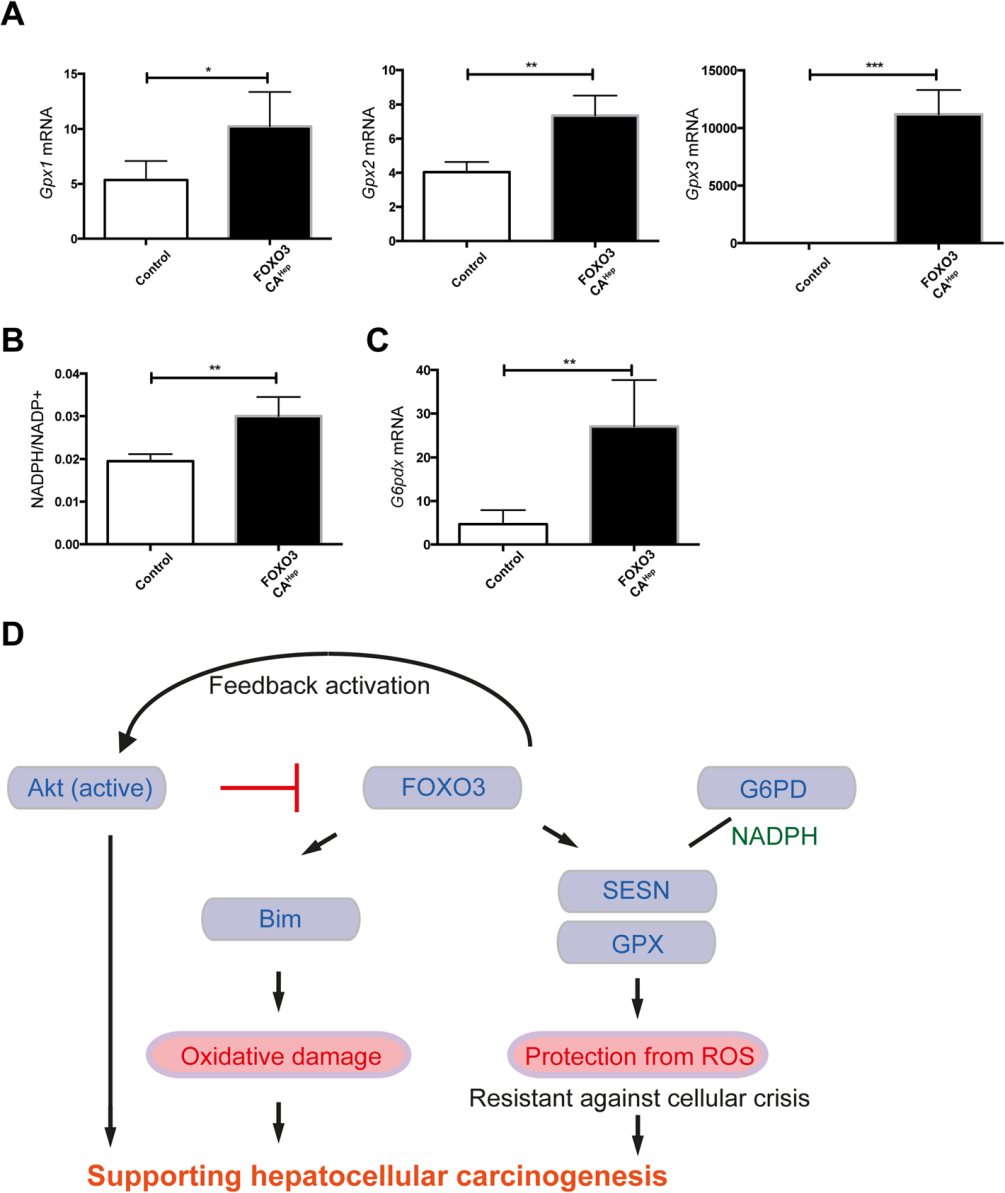
Hepatic activation of FOXO3 induces oxidative damage and protection system, and FOXO3 supports hepatocellular carcinogenesis in diverse ways. **a** Expression of *Gpx1*, *Gpx2*, and *Gpx3* by qPCR in livers from 9-week-old control and FOXO3CA^Hep^ transgenic mice (*n* = 4). **b** Higher NADPH/NADP+ ratio in 9-week-old FOXO3CA^Hep^ versus control mice. **c** Expression of *G6pdx* by qPCR in livers from 9-week-old control and FOXO3CA^Hep^ transgenic mice. **d** Graphical abstract. Here we propose that hepatic FOXO3 activates and balances both ROS-promoting and ROS-protecting pathways. On one side, hepatic activation of FOXO3 leads to oxidative damage and Akt activation, but on the other side, high expression of G6PD and NADPH, as well as glutathione peroxidases acting against oxidative damage. This balanced ROS control system presumably makes hepatocytes resistant against cellular death and crisis. The whole system can cooperatively support hepatocellular carcinogenesis

The role of FOXO3 in regulating ROS is still under debate. On one side, there are publications showing FOXO3 induces ROS, but on the other side, FOXO3 has been suggested to reduce ROS levels (e.g. [21],). In addition to high expression of peroxiredoxicin sestrins, we also observed high expression of glutathione peroxidases (*Gpx1*, *Gpx2*, *Gpx3*), which protect cells from oxidative damage (*n* = 4, *P* < 0.05, *P* < 0.01, *P* < 0.001, respectively) (Fig. 6a). NADPH is required for scavenging of ROS [22], and the NADPH/NADP+ ratio was higher in FOXO3CA^Hep^ mice (*P* < 0.01) (Fig. 6b). The major route for reduction of NADP+ to NADPH is the pentose phosphate pathway, where glucose-6-phosphate (G6PD) plays an important role as the rate-limiting enzyme of the pathway [23], and gene expression of *G6pdx* was elevated in FOXO3CA^Hep^ mice (*P* < 0.01) (Fig. 6c). In conclusion, hepatic FOXO3 activates and balances both ROS-promoting and ROS-protecting pathways. On the one hand, hepatic activation of FOXO3 leads to oxidative damage and Akt activation, but on the other hand, high expression of G6PD and NADPH, as well as glutathione peroxidases, act against oxidative damage. This balanced ROS-control system presumably makes hepatocytes resistant against cellular death and crisis. At the same time, it promotes hepatotoxicity-mediated hepatocellular carcinogenesis (Fig. 6d).

## Discussion

### FOXO3 can be both a positive and a negative regulator of ROS

In the current study, we identify a novel role of FOXO3 in controlling oxidative stress, which acts as a tumor promoter in hepatotoxin-induced tumor development. FOXO3 protein is overexpressed and activated in livers from HCC patients (Fig. 1a, b). However, we did not observe any significant difference of *FOXO3* mRNA expression between the non-HCC and the HCC samples (Fig. 1c). There is a discrepancy between *Foxo3* mRNA and protein expression in HCC tissues and non-HCC tissues. The precise mechanism of transcriptional regulation of *FOXO3* has not been fully understood. A study has suggested that the transcription of FOXO genes is stimulated by FOXO3 itself but repressed by growth factors in a cell line model [24]. A wide variety of growth factors are highly expressed in liver cancer [25], A mixture of several transcriptional stimulators and repressors may control *FOXO3* transcriptional regulation. Our observation about FOXO3 protein overexpression and activation in HCC samples nevertheless brought us a question, whether hepatic activation of FOXO3 in patients sufficiently supports hepatocellular carcinogenesis, or FOXO3 activation is just a consequence and secondary-effect during tumor development. In our transgenic mouse model, to that end we demonstrate that FOXO3 activation leads to the induction of several HCC-associated factors. Furthermore, hepatic activation of FOXO3 induces Akt activation via mTORC2 activation and supports hepatotoxicity-induced hepatocellular tumorigenesis. Several studies have suggested that FOXO3 is a positive regulator of ROS, but have also demonstrated as a negative regulator of ROS. Bim, a FOXO3-target is suggested to impair mitochondrial respiration and cause ROS production on the one hand [18]. On the other hand, FOXO3 activation causes mitochondrial gene expression and ROS reduction [21]. In the current study, we observed that FOXO3 induces both ROS-activating and ROS-inhibiting systems. Expression of Bim was upregulated and increased oxidative damage was observed in livers of FOXO3CA^Hep^ mice compared to control mice (Fig. 5a, b). We also observed a significantly higher expression of peroxidoxin sestrins and glutathione peroxidases in livers of FOXO3CA^Hep^ mice compared to control mice pointing to a protection of cells from oxidative damage (Figs. 5a and 6a). Therefore, we suggest that FOXO3 can either be a positive or a negative regulator of ROS in a context-dependent manner.

### FOXO3 can be both a tumor suppressor and a tumor promotor, but predominantly, FOXO3 supports cancer development

The role of FOXO3 in cancer has been intensively discussed. For a long time, FOXOs have been considered solely as tumor suppressors, since FOXOs are key transcription factors negatively regulated downstream from oncogenic PI3K/Akt. Furthermore, FOXOs are known to regulate a wide variety of target genes involved in, for example, cell cycle arrest and apoptosis, which may suppress tumor development. A knockout study also showed that loss of FOXOs enhances tumorigenesis in conditional knockout mice using the Mx1-Cre system [26]. However, increasing evidence demonstrates that high expression of FOXO3 is linked to a poor patient prognosis in several types of cancer. For example, high expression of FOXO3 is associated with a decreased overall survival in acute myeloid leukemia (AML) [27]. In pancreatic cancer patients, high FOXO3 activation signatures are associated with a poor prognosis through the promotion of cancer stem cell properties [11]. In addition, increased FOXO3 expression levels are associated with glioblastoma progression, mediated by autophagy and cell survival [10]. A recent study suggests that FOXO3 both suppresses and supports breast cancer progression [12]. We found that hepatic activation of FOXO3 leads to both accumulation and elimination of ROS. Therefore, stressed and damaged hepatocytes can still manage to escape from cellular crisis and death. These survived, damaged hepatocytes can undergo malignant transformation and support hepatotoxin-induced hepatocellular tumorigenesis (Fig. 3). FOXO3 is known to induce cell-cycle arrest, and in the current study, we did not observe any enhanced hepatocyte proliferation in 9-week-old FOXO3CA^Hep^ mice compared to control animals (data not shown). We have previously shown in another study that the inability of hepatocytes to initiate proper compensatory proliferation and inadequate liver regeneration can be associated with cellular damage and malignant transformation, leading to enhanced hepatocellular carcinogenesis [14]. We cannot exclude that cellular damage and malignant transformation of hepatocytes from FOXO3CA^Hep^ mice are caused by problems with the cell cycle. But, since FOXO3 is known to induce Bim, which has been suggested to impair mitochondrial respiration and cause ROS production, we suggest that the major reason for DNA damage in FOXO3CA^Hep^ mice is ROS.

### Pentose phosphate pathway and NADPH as supporters for protection from ROS and cellular crisis in hepatocytes of FOXO3CA^Hep^ mice

In FOXO3CA^Hep^ mice, we observed a significantly increased expression of peroxiredoxicin sestrins and glutathione peroxidases, which protect cells from oxidative damage (Figs. 5a and 6a). It has been shown that NADPH is required for scavenging of ROS [22], and in our study, hepatic activation of FOXO3 led to a significantly increased liver NADPH/NADP+ ratio (Fig. 6b). The major route for reduction of NADP+ to NADPH is the pentose phosphate pathway, where glucose-6-phosphate (G6PD) plays an important role as the rate-limiting enzyme of the pathway [23]. We also observed an elevated gene expression of *G6pdx* in livers of FOXO3CA^Hep^ mice (Fig. 6c), and we suggest that the pentose phosphate pathway plays an important role in supporting the ROS-protecting system in FOXO3CA^Hep^ mice. In line with this finding, it has been shown that inhibition of G6PD increases oxidative stress in breast cancer cells. Furthermore, high expression levels of G6PD are associated with a decreased overall relapse-free survival of breast cancer patients [28]. G6PD is suggested to be essential for cell survival [23] and inhibition of G6PD reduces proliferation and cell survival in breast cancer cells [28]. We assume that an elevated expression of G6PD and a higher NADPH/NADP+ ratio associated with the pentose phosphate pathway is able to support a ROS-preventing system and survival for ROS-mediated damaged hepatocytes in FOXO3CA^Hep^ mice.

### FOXO3 activates a positive feedback-loop for Akt signaling; parallel activation of PI3K/Akt and FOXO3 may be possible

We further demonstrate that hepatic activation of FOXO3 induces Akt activation (Fig. 5e, f). Under physiological conditions, active Akt negatively regulates FOXO3. Therefore, FOXO3 cannot be in an active form for a long time. However, we observed active FOXO3 in livers of several HCC patients (Fig. 1a, b). We hypothesize that FOXO3 stays active, even after PI3K/Akt signaling is activated. One explanation might be a FOXO3 mutation in HCC patients or patients with chronic liver disease (gain of function). A patient case with simultaneous mutations of all three Akt-phosphorylation sites (T32, S253, S315), like our mouse model, has not been reported yet, but a S253F Akt-phosphorylation site mutation has been identified in carcinoma patients [29, 30]. Another explanation involves reprogramming signal transduction systems. Akt is not the only negative regulator of FOXOs. SGK (serum/glucocorticoid regulated kinase), ERK, p38, and IKK are also suggested to inhibit FOXOs. On the other hand, JNK, Mst1, and AMPK are suggested to activate FOXOs [31]. Whether or not FOXO3 activation by these signaling systems can block or compete with Akt-mediated inhibition of FOXO3 needs to be further investigated.

### Personalized combination therapy targeting Akt and FOXO3 may be reasonable

With our data in mind, we would like to propose therapeutic options for HCC patients or for patients with chronic liver diseases. The PI3K/Akt signaling pathway is an attractive target in anti-cancer therapy, but unexpected combinational deletion of Akt1 and Akt2 in adult hepatocytes results in liver injury that promotes HCC in mice [32]. This is partially because long-term Akt inhibition can lead to chronic activation of FOXOs. In our study, we show that hepatic activation of FOXO3 induces a positive feedback loop for activating Akt (Fig. 5e, f). As we further demonstrated in the current study, activation of FOXO3 in hepatocytes not only activates Akt signaling, but also induces oxidative damage and survival of damaged cells.

## Conclusions

FOXO3 is a master regulator of ROS in a ‘carrot and stick’ manner; on one side avoiding cellular crisis while also supporting hepatocellular carcinogenesis. Clinically, we suggest analyzing FOXO3 activation status in patients with liver diseases, in addition to PI3K/Akt signaling. Akt inhibitor therapy remains an important option in cancer therapy. In the event that long term Akt therapy is needed, we would suggest additionally monitoring FOXO3 activity, and eventually offering combination therapy with PI3K/Akt inhibitors and FOXO3 inhibition.

## Data Availability

The data sets used and/or analyzed during the current study are available from the corresponding authors on reasonable request.

## Abbreviations

AFP: Alpha-Fetoprotein
ALT: Alanine aminotransferase
AML: Acute myeloid leukemia
AST: Aspartate transaminase
DEN: diethylnitrosamine
ELISA: Enzyme-linked Immunosorbent Assay
FOXO: Forkhead box O family
G6PD: Glucose-6-phosphate
HBV: Hepatitis B virus
HCC: Hepatocellular carcinoma
HCV: Hepatitis C virus
LAP: Liver-enriched transcriptional activator protein
MRI: Magnetic Resonance Imaging
NADP: Nicotinamide adenine dinucleotide phosphate
ROS: Reactive oxygen species
